# 4DCT Scanning Technique for Primary Hyperparathyroidism: A Scoping Review

**DOI:** 10.1155/2021/6614406

**Published:** 2021-05-21

**Authors:** Steven Raeymaeckers, Maurizio Tosi, Johan De Mey

**Affiliations:** ^1^Universitair Ziekenhuis Brussel, Laarbeeklaan 101, Jette 1090, Belgium; ^2^Radiology, Vrije Universiteit Brussel, Laarbeeklaan 103, Jette 1090, Belgium

## Abstract

**Objective:**

4DCT for the detection of (an) enlarged parathyroid(s) is a commonly performed examination in the management of primary hyperparathyroidism. Protocols are often institution-specific; this review aims to summarize the different protocols and explore the reported sensitivity and specificity of different 4DCT protocols as well as the associated dose.

**Materials and Methods:**

A literature study was independently conducted by two radiologists from April 2020 until May 2020 using the Medical Literature Analysis and Retrieval System Online (MEDLINE) database. Articles were screened and assessed for eligibility. From eligible studies, data were extracted to summarize different parameters of the scanning protocol and observed diagnostic attributes.

**Results:**

A total of 51 articles were included and 56 scanning protocols were identified. Most protocols use three (*n* = 25) or four different phases (*n* = 23). Almost all authors include noncontrast enhanced imaging and an arterial phase. Arterial images are usually obtained 25–30 s after administration of contrast, and less agreement exists concerning the timing of the venous phase(s). A mean contrast bolus of 100 mL is administered at 3-4 mL/s. Bolus tracking is not often used (*n* = 3). A wide range of effective doses are reported, up to 28 mSv. A mean sensitivity of 81.5% and a mean specificity of 86% are reported.

**Conclusion:**

Many different 4DCT scanning protocols for the detection of parathyroid adenomas exist in the literature. The number of phases does not appear to affect sensitivity or specificity. A triphasic approach, however, seems preferable, as three patterns of enhancement of parathyroid adenomas are described. Bolus tracking could help to reduce the variability of enhancement. Sensitivity and specificity also do not appear to be affected by other scan parameters like tube voltage or tube current. To keep the effective dose within limits, scanning at a lower fixed tube current seems preferable. Lowering tube voltage from 120 kV to 100 kV may yield similar image contrast but would also help lower the dose.

## 1. Introduction

Primary hyperparathyroidism is a common endocrine disease. In the case of an asymptomatic patient over the age of 50 without end-organ complications, conservative treatment can be assumed [1, 2]. The only cure for the disease is surgery, with resection of the affected gland(s). Bilateral exploration of the neck is the historical standard for treatment. In the last decades, however, a minimally invasive surgical approach has been made possible by more effective means of preoperative imaging combined with the development of rapid parathyroid hormone determination techniques allowing for intraoperative PTH monitoring [3].

The most accessible diagnostic technique is ultrasound because it is widely available at low cost, and it presents no adverse effects [4]. As a bonus, ultrasound is the preferred method of examination for the thyroid gland. This way parathyroid lesions can be differentiated from thyroid nodules and other thyroid pathologies. The sensitivity is very operator-dependent but can be as high as 84% in hands of an experienced ultrasonographer [5]. Color Doppler can be used to differentiate parathyroid lesions from other cervical masses, such as lymph nodes and thyroid nodules [6]. Small lesions (<5 mm) can be difficult to detect. False-negative results can occur, especially in the case of ectopic glands or in the presence of a large thyroid goiter [7].

Scintigraphy has the highest sensitivity compared to the other techniques: 88–90% and even higher sensitivity when combined with SPECT/SPECT-CT, the latter providing useful anatomical detail [8]. This technique is the method of choice when an ectopic localization is suspected or in patients having undergone prior neck surgery. The procedure relies on the uptake of 99mTechnetium (99mTc)-sestamibi in the overactivated mitochondria-rich oxyphil cells of the parathyroid gland and the difference in the rate of washout of this tracer with the thyroid. To overcome false positive results deriving from the uptake of sestamibi in solid thyroid nodules, another tracer can be administered that is exclusively taken up by the thyroid tissue, thereby permitting a subtraction of the thyroid. This method is known as the double-tracer technique and can achieve a specificity of over 90% [9].

MRI also allows for the evaluation of parathyroid disease. This technique, like ultrasound, is free of ionizing radiation but it is far a less available modality (a problem also found with scintigraphy). Due to the lack of ionizing radiation, MRI can be used without hesitation to detect ectopic glands. On a 1.5 Tesla system, the reported sensitivity of this technique is 80%; assumedly a better visualization on 3 Tesla systems can be obtained [10–12].

Computed tomography (CT) of the neck without contrast is of no value since it is not possible to discern between parathyroid tissue, ectopic thyroid tissue, and lymph nodes. Four-dimensional CT (4DCT) combines three-dimensional imaging with the inclusion of time as the fourth dimension: this allows for an evaluation of the pattern of enhancement of lesions over time. By means of the evaluation of enhancement, abnormal parathyroid glands can be detected with a sensitivity of 85.7%: typical parathyroid adenomas are hypoattenuating to thyroid tissue on noncontrast enhanced imaging (NECT) and demonstrate avid arterial hyperenhancement during the arterial phase as well as rapid washout on the venous phase [13, 14]. 4DCT is also considered to be a useful technique in the case of ectopic glands or in the case of persistence/recurrence after the initial surgery.

Many different study protocols have been suggested by different authors over the past 15 years; protocols are often very institution-specific. We set out to examine the literature to review the different protocols and their reported sensitivity/specificity. The number of different phases used in a protocol as well as their timing could be a defining factor in sensitivity: as more phases are available, it could be argued that this would increase the sensitivity of the examination. Factors like tube current, tube voltage, and administration of contrast medium affect image noise and contrast and could therefore also have implications toward sensitivity. These factors also influence the effective dose, a key part to consider in multiphasic studies using ionizing radiation.

## 2. Materials and Methods

### 2.1. Research Question

This scoping review seeks to answer a multivalent research question. First, we want to establish the number of phases and their timing as used by different authors in the literature. Secondly, we wish to assess whether different protocols are associated with different sensitivity or specificity. We also look at other scanning protocol factors like tube current and tube voltage, contrast bolus volume, and timing: factors that not only can affect the sensitivity of the exam but also influence the effective dose, a key part to consider in multiphasic studies using ionizing radiation.

### 2.2. Search Strategy

This scoping review is reported according to the PRISMA Extension for Scoping Reviews (PRISMA-ScR) Checklist. A literature search was independently conducted by two radiologists using the MEDLINE database from April 2020 until May 2020 using the terms “4DCT” and “parathyroid.”

### 2.3. Inclusion and Exclusion Criteria

We included all peer-reviewed literature written in English or French. There was no limitation on the publication date. Exclusion criteria were articles written in a language other than English or French.

### 2.4. Data Selection and Charting Process

The two investigators independently screened the MEDLINE database for relevant articles. A first selection was made based on the title and abstract. Next, each investigator screened the obtained articles for relevancy, and a second selection was made. Finally, the remaining articles underwent a final screening: only studies citing information about the number of phases used in the scanning protocol were included in the scoping review. Forty articles were not relevant. Information regarding publication details (e.g., author, publication date) and study details (e.g., study design, scanning protocol) was obtained. Data was managed in Excel (Microsoft, Redmond, USA). Several scanning protocol factors were included in this review. We checked for the presence of a NECT in the study protocol. We evaluated the number of obtained phases and their timing: we defined a subdivision in an arterial phase (earlier than 40 seconds after contrast administration), a venous phase (40 seconds–70 seconds), a delayed venous phase (70 seconds–100 seconds), and a very delayed phase (later than 100 seconds). We evaluated whether this timing was expressed in absolute or relative relation to the onset of contrast administration; we expressed (where possible) all times in absolute relation to the administration of contrast. We also checked for the use of bolus tracking, which is defined as the use of a continuous density measurement in an arterial structure (usually at the level of the aortic arch) prior to obtaining the first arterial phase.

We also included other parameters of the scanning protocol: tube current is determined by the rate at which X-rays are produced in the X-ray tube (i.e., photons per second). It is expressed in milliampere (mA). Tube voltage is a parameter in direct relation to the number of X-rays produced. It is expressed in kilovolt (kV). The volume of the contrast bolus was included, as well as the injection speed expressed in mL/s. We included the effective dose, expressed in millisievert (mSv). Lastly, we included the reported sensitivity, PPV, specificity, and other related data provided.

## 3. Results

### 3.1. Identification of Potential Studies

**Figure 1:**
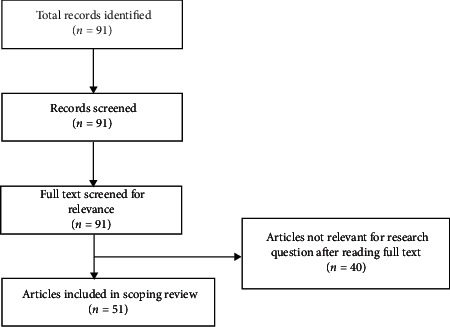
Flow chart of the studies' selection process.

The searches from the MEDLINE database hit a total of 91 records that were screened after the removal of duplicates. All articles were available to us in full-text format. The full-text screening stage led to 91 potential articles relevant to our scoping review. Additional articles were excluded after full-text assessment for the reasons mentioned in “Materials and Methods”. A flow chart is provided in Figure 1.

A total of 51 articles were included, as the authors of these had defined the number of phases and at least some of the parameters of the scanning protocol. These articles are listed chronologically in order of most recently published in Table 1. In the case of six studies, the authors defined two different protocols, thus amounting to 56 scanning protocols. These alternative protocols were added in Table 1 as secondary rows in relation to the first protocol.

### 3.2. Characteristics of the Included Studies

The peer-reviewed literature on the subject of 4DCT for the detection of parathyroid adenomas is recent: the concept of 4DCT for the detection of parathyroid adenomas was coined in 2006 by Rodgers et al. Among the included studies, 45% were published in the last five years. Most studies (76.5%) are retrospective studies.

### 3.3. Number of Phases

Most authors use a triphasic approach: 25 study protocols were described in this manner. A four-phasic approach is almost as prevalent; 23 study protocols were described in this manner. Far less common are biphasic studies: only 7 of these protocols could be identified. One study was unique since the authors made use of a five-phasic approach.

### 3.4. Use of a Noncontrast Enhanced Phase

Fifty-two out of 56 study protocols include a noncontrast enhanced phase (NECT), before administration of contrast medium. Only 4 protocols were identified in which the authors did not acquire a NECT; these protocols were all two-phasic in nature.

### 3.5. Contrast Phase Timing and Bolus Tracking

Only three authors use bolus tracking. All other authors opt for a fixed time interval after the start of contrast bolus administration before obtaining the first contrast phase, almost always an arterial phase.

**Figure 2:**
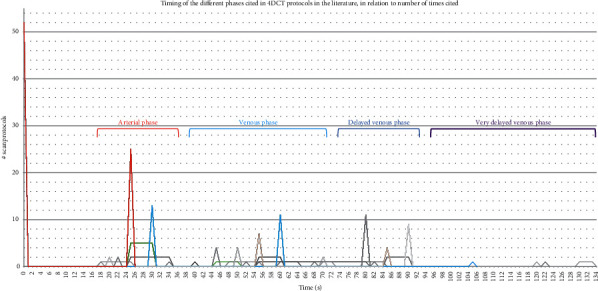
Timing of the different phases of 4DCT protocols in the literature, in relation to the number of citations for each of these relevant time points.

Many authors do not provide definitions for the later phases in absolute terms (relation to the start of contrast bolus administration) and refer to relative intervals between the different phases. Since many authors do not describe pitch and rotation speed or refer to an exact scan length, the scan time of the different phases themselves is often unclear. This further complicates the definition of an exact timeframe. We expressed (where possible) all times in absolute relation to the onset of contrast administration; some error can be expected on a conversion. A graphic overview of the timing of the different phases of the selected studies is presented in Figure 2; this is in relation to the number of protocols citing these relevant time points.

### 3.6. Use of an Arterial Phase

Except for four studies, all cited studies choose to obtain an arterial phase. In the case of 25 different studies, the arterial images were obtained 25 s after contrast administration; in 13 studies, the authors proposed scanning after 30 s. Five other authors stated that the arterial images were obtained by scanning after 25–30 s. Forty-two protocols then perform arterial scanning after 25 to 30 s.

### 3.7. Use of Other Phases

The venous phase is obtained most often and can be acquired as early as 45 seconds up to 70 seconds after administration of contrast. The delayed venous phase is less popular and is usually acquired between 70 seconds and as late as 90 seconds after administration of contrast. Rarely, authors also acquire an even more delayed phase: later than 100 seconds after bolus administration and even up to 130 seconds.

### 3.8. Contrast Administration

Most studies define the used contrast medium, bolus volume, and injection speed. A mean volume of 100 mL contrast is administered, most commonly at 3 or 4 mL/s. Two authors define the contrast volume in function of the patient's weight.

### 3.9. Effective Dose

Only 24 authors present data concerning the effective dose. The described doses are wide-ranging, with the lowest dose reported at 5.5 mSv and an upper limit of 28 mSv. The mean effective dose for all relevant studies combined is calculated at 15.96 mSv. 27 authors do not discuss dose limits.

The mean effective dose for the three biphasic studies with available data is calculated at 8.1 mSv. For the three- and four-phasic studies, the mean effective dose is higher, 18.1 mSv and 15.4 mSv, respectively. The authors of the only five-phasic study report an effective dose range of 5.6–10.4 mSv.

### 3.10. Appraisal of Sensitivity/Specificity

All included studies were assessed for the quality of the reported findings. 74.5% of the included studies report on sensitivity and specificity. Some studies (14%) do not refer to general sensitivity but report on the sensitivity of lateralization or correct selection of the diseased quadrant.

We find a mean sensitivity (*n* = 23) of 81.5% for the detection of parathyroid adenomas using 4DCT. Only two studies report a lower sensitivity of 50%; both studies are four-phasic studies. We find a mean PPV (*n* = 17) of 90%. Less data is available on specificity (*n* = 11), and the mean specificity is calculated at 86%.

## 4. Discussion

**Figure 3:**
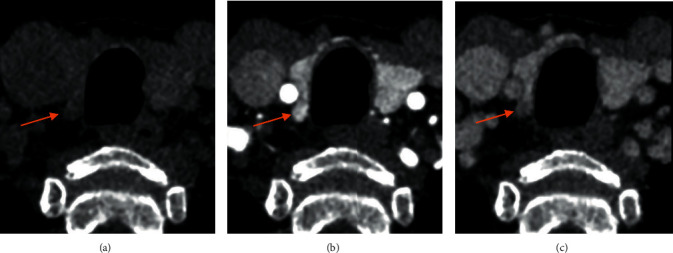
4DCT of a small right upper parathyroid adenoma. (a) NECT with lower attenuation (b) Arterial phase with higher enhancement. (c) Venous phase with lower enhancement.

Typical parathyroid adenomas are hypoattenuating to thyroid tissue on NECT and demonstrate avid arterial enhancement during the arterial phase as well as rapid washout on the venous phase. The arterial phase is the most important, as adenomas can be visualized as hyperenhancing nodules in characteristic locations. In Figure 3, we provide an example of a parathyroid adenoma behaving accordingly during all three phases. Unfortunately, for approximately one-third of adenomas, the lesion will prove to be isoattenuating to the thyroid on arterial and venous phases [15].

Most authors agree to include a noncontrast enhanced phase. This phase does seem to be important in the diagnosis of parathyroid adenomas, as the study by Bahl et al. [38] identifies a type C pattern of enhancement. These lesions mimic thyroid tissue and can only be discerned by the use of noncontrast-enhanced imaging since these lesions should be lower in density compared to the thyroid on the noncontrast enhanced series. Almost all authors choose to include an arterial phase. According to Bahl et al., this is necessary to diagnose type A lesions, which demonstrate a higher peak enhancement compared to the thyroid in the arterial phase. A venous or delayed venous phase is commonly used. According to Bahl et al., this is necessary to diagnose type B lesions, which demonstrate a lower enhancement compared to the thyroid in the venous phase.

Most authors opt for a delay of 25 up to 30 seconds for the arterial phase after contrast administration. There is more variance concerning the timing of the venous phases, with some authors scanning as early as 40 seconds after administration of contrast and others no less than 50 seconds later. Other authors even prefer very delayed venous phases. We find no difference regarding the sensitivity or specificity of 4DCT in comparison to the number of phases or their timing. The two studies with the lowest sensitivity are both four-phasic studies, with numerous three-phasic studies reporting higher sensitivity.

Only three authors use bolus tracking to optimize the scanning delay, although this could easily be implemented. If the arterial peak enhancement of the adenoma should prove to be a short-lived effect, scanning at a fixed interval of time might risk missing the diagnosis. This would prove especially true in older patients with suboptimal cardiac output, as the arterial phase could be performed too early in these cases. Bolus tracking at the level of the aortic arch would allow for the arterial and later phases to be obtained at a variable time interval, with reduced variability of arterial enhancement and less risk of scanning prematurely. Bolus tracking is commonly used in clinical practice, without significant dose effect [64].

Regarding contrast administration, the variance between the reviewed studies is limited. Administered bolus volumes are situated between 75 mL and 120 mL with a mean volume of 100 mL. Injection speed varies mostly between 3 and 4 mL/s. A contrast bolus with a lower volume or at a lower injection speed may affect lesion enhancement and the timing of said enhancement. No difference regarding sensitivity or specificity of 4DCT is found, however, for different volumes or injection speeds.

What proves striking is that 27 out of 51 authors do not mention effective dose when discussing a multiphasic CT scan of the neck. The mean effective dose for the 24 studies that do comment on patient dose is calculated at 15.96 mSv. The described dose limits are wide-ranging, with an upper limit of 28 mSv. These upper values should be considered as very high and place direct limits on the number of phases. As a limitation, it should be noted that it is difficult to calculate a correct effective organ dose. The presence of an iodinated contrast medium is a known factor that increases organ dose, yet this has not been factored into any known conversion method [65].

The mean effective dose is the lowest for biphasic studies, as could be expected. The mean effective dose for the three studies with available effective dose data is calculated at 8.1 mSv. For the more popular three- and four-phasic studies, the mean effective dose is higher, but the mean effective dose for the four-phasic studies appears lower in comparison to the three-phasic studies (15.4 mSv versus 18.1 mSv). This is probably because of lower tube current settings, an effect that can also be observed in the only five-phasic study. Here, a low mean effective dose range of 5.6–10.4 mSv is reported, probably because the authors do not use automatic tube current modulation but instead opt for a fixed tube current of 200 mA.

Changes in tube current will affect image noise: higher mA settings will result in lower noise. Changes in tube voltage will affect the amount of contrast in the resultant image: higher kV settings will result in an increase in contrast. Since increasing the tube current improves image quality by reducing noise without affecting image contrast, it can be argued that to keep the effective dose for classic 4DCT within acceptable levels, a lower fixed tube current should be preferred. Most protocols utilize a 120 kV tube voltage. Since diagnosis relies primarily on enhancement after contrast, it can be suggested that scanning at 100 kV tube voltage would yield similar image contrast, but at a lower effective dose [66]. Again, regarding tube parameters, no difference in sensitivity or specificity is found for different values.

## 5. Conclusion

Many different 4DCT scanning protocols for the detection of parathyroid adenomas exist in the literature. We find a mean sensitivity of 81.5% and a mean specificity of 86%. The number of phases does not appear to affect sensitivity or specificity. A triphasic approach, however, seems preferable, as three patterns of enhancement of parathyroid adenomas are described and require nonenhanced imaging as well as an arterial and a venous series. Arterial images are usually obtained 25–30 s after administration of contrast, and less agreement exists concerning the timing of the venous phase(s). A mean contrast bolus of 100 mL is administered at 3–4 mL/s. Bolus tracking is not often used but could help to reduce the variability of enhancement. Sensitivity and specificity do not appear to be affected by other scan parameters like tube voltage or tube current. To keep the effective dose within limits, scanning at a lower fixed tube current seems preferable. Lowering tube voltage from 120 kV to 100 kV may yield similar image contrast but would also help lower the dose.

## Figures and Tables

**Table 1:** Overview of the different protocols in the literature: number and timing of different phases, tube current/voltage, contrast volume and injection speed, effective dose, and sensitivity/specificity/PPV/NPV/accuracy.

Reference	Authors	Year published	Study design	NECT	Tracking	Contrast phase timing	Number of phases	Arterial phase	Venous phase	Delayed phase	Very delayed phase	Tube current (mA)	Tube voltage (kV)	Contrast volume	Injection speed	Effective dose (mSv)	Sensitivity/specificity/PPV/NPV/accuracy
[15]	Vijayasarathi et al.	2020	Case series	Yes	Yes	Relative	3	25–30 s	60–80 s	—	—	?	?	?	?	?	

[16]	Wojtczak et al.	2020	Review	Yes	No	Relative	4	25–30 s	25–30 s	85–90 s	—	?	?	?	?	?	

[17]	Acar et al.	2020	Retrospective study	Yes	No	Absolute	4	25 s	40 s	80 s	—	?	?	0.1 mL/kg	?	?	Sensitivity 50%
																	Specificity 100%
																	PPV 100%
																	NPV 85%
																	Accuracy 86.9%

[18]	Zafereo et al.	2019	Review	Yes	No	Relative	3	25–30 s	55–60 s	—	—	?	?	?	?	27–28	
				Yes	No	Relative	3	25–30 s	—	70 s	—	?	?	?	?	27–28	

[19]	Kedarisetty et al.	2019	Retrospective study	Yes	No	Absolute	3	25 s	—	85 s	—	?	?	75 mL	?	?	Sensitivity 80%
																	Specificity 75%
																	PPV 92%
																	NPV 50%
																	Accuracy 79%

[20]	Yeh et al.	2019	Retrospective study	Yes	No	Relative	3	30 s	60 s	—	—	?	?	75 mL	4 mL/s	22	Sensitivity 79%
																	Specificity 96%
																	PPV 90%
																	NPV 90%

[21]	Amadou et al.	2019	Retrospective study	Yes	No	Absolute	3	—	45 s	70 s	—	50–100	120	120 mL	?	10–27	Sensitivity 75%
																	Specificity 40%
																	PPV 80%
																	NPV 33%

[22]	Vu et al.	2019	Retrospective study	Yes	No	Absolute	4	25 s	55 s	85 s	—	220	130	120 mL	4 mL/s	17.9	

[23]	Binks et al.	2019	Retrospective study	Yes	No	Absolute	3	25–30 s	45–50 s	—	—	?	?	?	?	7.99–28	Sensitivity 85.7%
																	PPV 94.7%

[24]	Cunha-Bezerra et al.	2018	Retrospective study	Yes	Yes	Relative	4	18–25 s	48–55 s	?	—	120–200	120	2 mL/kg max 120 mL	4-5 L/s	?	

[25]	Tian et al.	2018	Retrospective cohort study	Yes	Yes	Relative	3	Aorta: 150HU	30 s later	—	—	?	?	100 mL	4 mL/s	9.3	Sensitivity 72.9%
																	Sensitivity 60% (persistent/recument disease)
																	Sensitivity 67.4% (multigland disease)

[26]	Christakis et al.	2017	Retrospective study	Yes	No	Absolute	4	25 s	?	?	—	?	?	120 mL	3 mL/s	?	Sensitivity 79%
																	Specificity 100%
																	PPV 100%
																	NPV 44%
																	Accuracy 82%

[27]	Morón et al.	2017	Retrospective study	Yes	No	Absolute	2	25 s	—	—	—	150–600	120	100 mL	4 mL/s	?	Sensitivity 80%
																	Specificity 97%
																	PPV 89%
																	Accuracy 92%

[28]	Goroshi et al.	2017	Retrospective study	Yes	No	Absolute	4	20 s	60 s	90 s	—	140–220	120	60 mL	4 mL/s	18.9	Left/right localization: Sensitivity 93% (hypercalcemic)
																	Quadrant localization: Sensitivity 90% (hypercalcemic)

[29]	Taywade et al.	2017	Prospective study	Yes	No	Absolute	3	25 s	—	80 s	—	?	?	75 mL	4 mL/s	11–13	

[30]	Zeina et al.	2017	Prospective study	Yes	No	Absolute	4	25 s	60 s	90 s	—	400	120	120 mL	4 mL/s	?	Accuracy 96%

[31]	Sho et al.	2016	Prospective study	Yes	No	Relative	3	25 s	55 s	—	—	230	120	100–120 mL	3-4 mL/s	?	Sensitivity: 79% (multigland disease)
																	Specificity: 63%(multigland disease)
																	PPV:58% (multigland disease)

[32]	Fitzgerald et al.	2017	Retrospective study	Yes	No	Absolute	3	30 s	—	90 s	—	180–600	120	75 mL	3 mL/s	10.4–28	

[33]	Rameau et al.	2017	Case series	Yes	No	Relative	3	25 s	55 s	—	—	150–300	120	100–120 mL	4 mL/s	?	Sensitivity 84.6%
																	PPV 91.7%

[34]	Ramirez et al.	2016	Retrospective study	Yes	No	Absolute	4	25 s	55 s	85 s	-	?	?	120 mL	4 mL/s	14	Sensitivity 93%
																	PPV:96%
				Yes	No	Absolute	2	25 s	—	—	—	?	?	120 mL	4 mL/s	6.8	Sensitivity 97%
																	PPV:94%

[35]	Forghani et al.	2016	Retrospective study	Yes	No	Absolute	4	25 s	55 s	85 s	—	?	?	100 mL	3.5 mL/s	2.4–4.1	

[36]	Lee et al.	2016	Retrospective study	Yes	No	Absolute	4	30 s	60 s	90 s	—	140–240	120	90 mL	5 mL/s	10.9	

[37]	Hinsin et al.	2015	Case series	Yes	No	Absolute	4	30 s	—	80 s	—	?	?	75 mL	3 mL/s	13.8	Left/right localization: Sensitivity 84.2% (hypercalcemic)
																	Left/right localization: Specificity 81.8% (hypercalcemic)
																	Quadrant localization: Sensitivity 76.5% (hypercalcemic)
																	Quadrant localization: Specificity 91.59% (hypercalcemic)

[38]	Bahl et al.	2015	Retrospective study	Yes	No	Absolute	3	25 s	45 s	70 s	—	100–700	120	75 mL	4 mL/s	28	Sensitivity 85%
																	Sensitivity: 94% (single gland disease)
																	Sensitivity: 59% (multigland disease)

[39]	Boury	2015	Review	Yes	No	Absolute	3	—	45 s	70 s	—	?	?	?	3 mL/s	?	

[40]	Hoang et al.	2015	Phantom study	Yes	No	Absolute	3	25 s	—	80 s	—	100–500	120	75 mL	4 mL/s	28	

[41]	Lundstroem et al.	2016	Retrospective study	Yes	No	Absolute	5	22 s	52 s	82 s	122 s	200	120	100 mL	3.5 mL/s	5.56–10.4	Left/right localization: Sensitivity 84% (hypercalcemic)
																	Left/right localization: Specificity 76% (hypercalcemic)
																	Quadrant localization: Sensitivity 55% (hypercalcemic)
																	Quadrant localization: Specificity 50% (hypercalcemic)

[42]	Seeliger et al.	2015	Prospective study	Yes	No	Absolute	4	25 s	50 s	80 s	—	250	120	120 mL	2 mL/s	?	PPV 93%

[43]	Cham et al.	2015	Retrospective study	Yes	No	Relative	3	25–34 s	55–64 s	—	—	230	120	100–120 mL	?	?	

[44]	Day et al.	2015	Retrospective study	Yes	No	Absolute	4	30 s	60 s	90 s	—	100–750	120	100,L	3 mL/s	?	Sensitivity 93%
																	PPV 80%

[45]	Campbell et al.	2015	Retrospective study	Yes	No	Absolute	2	—	50 s	—	—	100–440	120	75–100 mL	?	5.2	Left/right localization: Sensitivity 77% (hypercalcemic)
																	Left/right localization: Specificity 87% (hypercalcemic)
																	Quadrant localization: Sensitivity 58% (hypercalcemic)
																	Quadrant localization: Specificity 91% (hypercalcemic)

[46]	Sepahdari et al.	2015	Retrospective study	Yes	No	Absolute	3	25 s	55 s	—	—	230	120	100–120 mL	3–4 mL/s	?	Sensitivity 77%
				Yes	No	Absolute	3	25 s	—	80 s	—	230	120	100–120 mL	3–4 mL/s	?	Sensitivity: 95% (single gland disease)
																	Sensitivity: 55% (multigland disease)

[47]	Suh et al.	2015	Case series	Yes	No	Absolute	4	30 s	60 s	90 s	—	140–240	120	90 mL	?	?	Sensitivity 92.1%
																	Specificity 95.6%
																	PPV 87.5%
																	NPV 97.3%
																	Accuracy 94.7%

[48]	Ginsburg et al.	2015	Retrospective study	Yes	No	Absolute	4	25 s	55 s	85 s	—	200	120	120 m	4 mL/s	?	Sensitivity 50%
																	PPV 100%
																	Accuracy 54.5%

[49]	Raghavan et al.	2014	Retrospective study	Yes	No	Absolute	4	25 s	50 s	80 s	—	50–400	120	120 mL	4 mL/s	?	*Accuracy, sensitivity, and specificity for localization and lateralization of the arterial phase alone were comparable with the full complement of 4 phases*

[50]	Brown et al.	2015	Retrospective cohort study	Yes	No	Absolute	3	34 s	68 s	—	—	?	?	70 mL	3 mL/s	7.99 mSv (3 phases)	Sensitivity 92%
																10.88,Sv(4 phases)	PPV 89%

[14]	Hoang et al.	2014	Case series	Yes	No	Absolute	3	25 s	—	80 s	—	100–400	120	75 mL	4 mL/s	?	

[51]	Hunter et al.	2014	Retrospective study	Yes	No	Relative	3	30 s	60 s	—	—	180–300	140	100 mL	4 mL/s	?	Sensitivity 94%
																	Specificity 98.3%
																	PPV 96.7%
																	NPV 97.1%

[52]	Bahl et al.	2014	Retrospective study	No	No	Absolute	2	20 s	70 s	—	—	100–400	120	100 mL	4 mL/s	?	
				Yes	No	Absolute	3	25 s	—	80 s	—	100–700	120	75 mL	4 mL/s	?	

[53]	Kely et al.	2014	Retrospective study	Yes	No	Relative	4	30 s	45 s	90 s	—	180–300	140	100 mL	4 mL/s	28	PPV 85.8%
																	Accuracy 77.9%
				Yes	No	Relative	3	30 s	45 s	—	—	180–300	140	100 mL	4 mL/s	21	PPV 89%
																	Accuracy 87.1%

[54]	Sepahdari et al.	2013	Retrospective study	Yes	No	Relative	3	25–34 s	55–84 s	—	—	230	120	100–120 mL	3–4 mL/s	2.2 mSv/phases	*Interobserver comparison*
																	Sensitivity 73% vs 55%
																	Specificity 88% vs 100%
																	PPV 91% vs 100%
																	NPV 64% vs 52%
																	Accuracy 78% vs 70%

[55]	Hunter et al.	2012	Retrospective study	Yes	No	Relative	4	25 s	70–73 s	—	130–133 s	240	140	120 mL	4 mL/s	21 mSv (3 phases)	Left/right localization: Accuracy 93.7%
																27 mSv (4 phases)	Quadrant localization: Accuracy 86.6%

[56]	Mahajan et al.	2012	?	Yes	No	Absolute	4	30 s	60 s	90 s	—	?	?	80 mL	?	10.4	

[57]	Gafton et al.	2012	Case series	No	No	Absolute	2	25 s	—	80 s	—	?	120	120 mL	3 mL/s	?	

[58]	Kutler et al.	2011	Retrospective study	Yes	No	Absolute	2	—	50 s	—	—	220–320	120	125 mL	3 mL/s	12.2	

[59]	Eichhom-Wharry et al.	2011	Retrospective study	No	No	?	2	18 s	Immediately	—	—	?	?	100 mL	?	?	Accuracy 73%
				No	No	?	2	22 s (>55y)	Immediately	—	—	?	?	100 mL	?	?	

[13]	Starker et al.	2011	Retrospective study	Yes	No	Absolute	4	30 s	60 s	—	120 s	100–750	120	100 mL	3 mL/s	?	Left/right localization: Sensitivity 93.9%
																	Left/right localization: PPV 93.5%
																	Quadrant localization: Sensitivity 85.7%
																	Quadrant localization: PPV 84.9%

[60]	Beland et al.	2011	Retrospective study	Yes	No	Absolute	4	30 s	60 s	90 s	—	100–750	120	?	?	?	Sensitivity 82%
																	Specificity 92%

[61]	Lubitz et al.	2010	Retrospective study	Yes	No	Relative	4	30 s	60 s	—	105 s	180–300	140	100 mL	?	10	Left/right localization: Sensitivity 73%
																	Quadrant localization: Sensitivity 60%

[62]	Mortenson et al.	2008	Retrospective study	Yes	No	Relative	4	25 s	55 s	85 s	—	?	?	120 mL	4 mL/s	?	Sensitivity 88%

[63]	Rodgers et al.	2006	Mixed prospective and retrospective study	Yes	No	?	3	25 s	?	—	—	220	140	120 mL	3 mL/s	?	Sensitivity 88%
																	Quadrant localization: Sensitivity 70%

## Data Availability

No data were used to support this study.
